# Bidirectional portal vein reconstruction for type C anatomy in living donor liver transplant

**DOI:** 10.3389/ti.2026.16600

**Published:** 2026-07-13

**Authors:** Shams-Ud-Din, Bigyan Acharya, Abdul Wahab Dogar, Mohammad Arsalan, Muhammad Umar, Syed Hasnain Abbas

**Affiliations:** Department of Hepatobiliary and Liver Transplant Surgery, Gambat Institute of Medical Sciences (GIMS), Gambat, Sindh, Pakistan

**Keywords:** dual-patch venoplasty, living donor liver transplantation, portal inflow optimization, portal vein reconstruction, type C portal vein anatomy

Dear Editors

Portal vein (PV) reconstruction remains a critical technical challenge in living donor liver transplantation (LDLT), particularly in the presence of complex anatomical variants such as Type C portal vein anatomy, characterized by multiple portal branches without a dominant trunk [1–3]. These configurations are associated with size mismatch, spatial separation, and unfavourable inflow geometry, increasing the risk of thrombosis and anastomotic stenosis.

Autologous Y-graft interposition has traditionally been used to reconstruct multiple PV branches and has demonstrated acceptable outcomes [4, 5]. However, this approach introduces additional anastomoses and may result in geometric distortion, turbulence, and functional narrowing, particularly in complex anatomical settings and Conjoined unification venoplasty (CUV) was subsequently developed to create a single wide conduit by unifying multiple graft PV branches, thereby improving inflow alignment and reducing anastomotic complexity [6–8]. Despite these advantages, graft-side reconstruction alone may not adequately address recipient PV limitations, especially in cases with a narrow or unfavorable recipient PV.

We report our experience with a bidirectional dual-patch PV reconstruction technique (Figure 1) that simultaneously optimizes graft and recipient PV geometry in LDLT with Type C anatomy. This approach combines graft conjoined unification venoplasty using a rectangular autologous PV patch with recipient PV augmentation using a triangular patch, enabling a wide, tension-free, and size-matched anastomosis without the need for interposition grafts.

**FIGURE 1:**
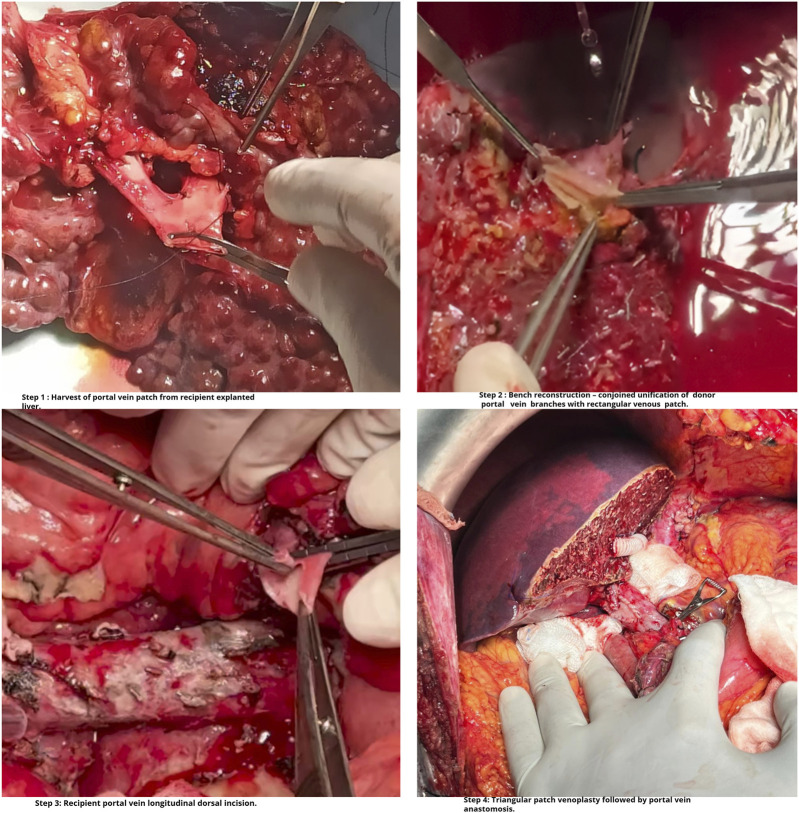
Surgical steps of bidirectional dual-patch PV reconstruction.

Between January 2016 and January 2026, 1,385 LDLTs were performed at our center. Type C PV anatomy was identified in 32 donors (2.31%), of whom 25 required PV reconstruction. 16 patients underwent portal vein reconstruction using autologous Y-graft interposition,5 consecutive patients underwent bidirectional dual-patch reconstruction and constitute this series, Direct venoplasty was feasible in 4 cases due to proximity of portal vein branches & rest of the 7 cases where left lateral graft were taken where no reconstruction were needed. Patients with pre-existing PV thrombosis were excluded.

Our technique for bidirectional dual-patch PV reconstruction (Figure 1) is following recipient hepatectomy, an autologous PV segment was harvested from the explanted liver. On the back table, a rectangular patch was used to unify adjacent graft PV branches into a single enlarged orifice. The recipient PV was then augmented with a longitudinal incision and triangular patch venoplasty to increase luminal diameter and accommodate the reconstructed graft PV. End-to-end anastomosis was performed with continuous sutures, ensuring proper alignment and absence of tension. Intraoperative Doppler ultrasonography confirmed adequate portal inflow.

PV reconstruction was successfully completed in all patients without interposition grafts. No early PV thrombosis or clinically significant stenosis occurred. All recipients demonstrated satisfactory graft function, and PV patency was maintained during 6 months follow-up, with physiologic Doppler flow (mean velocity approximately 35 cm/s).

In contrast, in our limited experience of portal vein reconstruction using autologous Y-graft interposition in 16 cases, size mismatch, conduit buckling, and angulation-related inflow compromise emerged as the principal technical challenges. These complications necessitated surgical re-anastomosis in 3 cases (18.75%) and endovascular stenting in 1 case (6.25%). While Y-graft interposition remains an effective reconstructive strategy, it is technically demanding during bench preparation and portal vein reconstruction. Particularly in patients with chronic portal vein thrombosis, distorted portal venous morphology, and small-caliber main portal veins where Y-graft interposition may be technically challenging. This inherent fixed anatomical configuration may predispose to angulation-related inflow disturbances, potentially resulting in flow impairment and postoperative thrombosis, thereby requiring re-intervention in a subset of patients. These findings are comparable with previously reported series [6]. Although conjoined unification venoplasty (CUV) improves graft-side configuration, the resulting conduit may exceed the adaptive capacity of the recipient PV and wider common channel. These observations emphasize that successful PV reconstruction depends not only on graft-side unification but also on appropriate recipient PV accommodation. Asan medical center came up with conjoined unification venoplasty (CUV) method in 2014 which is a technical modification of conventional autologous Portal vein Y graft interposition introduced in 2001 [6–8]. To overcome these issues we came up with bidirectional dual-patch PV reconstruction.

However, the bidirectional dual-patch technique addresses both components of this problem by combining graft unification with controlled recipient PV enlargement we achieved adequate diameter of main PV, this strategy facilitates a harmonized inflow pathway with improved geometric alignment. This is particularly relevant in LDLT, where partial grafts are exposed to increased portal inflow and are sensitive to disturbances in flow dynamics. Maintenance of a laminar, low-resistance inflow is essential to preserve endothelial integrity and support graft function [6–8].

In this small series, the absence of PV-related complications and sustained patency suggest that this technique is safe and reproducible. Although the number of patients is limited and follow-up duration is relatively short, the consistent hemodynamic performance supports further evaluation of this approach in larger cohorts. We are currently extending this experience through a comparative study evaluating the bidirectional dual-patch technique against Y-graft interposition to further define its relative advantages.

In conclusion, bidirectional dual-patch PV reconstruction represents a practical and physiologically sound option for managing Type C PV anatomy in LDLT. By restoring ideal graft–recipient portal venous geometry while eliminating the need for interposition grafts, this technique minimizes anastomotic complexity and has the potential to reduce flow-related complications. It should be considered a strong alternative to Y-graft interposition, and warrants further validation in larger, comparative studies with long-term follow-up.

## Data Availability

The original contributions presented in the study are included in the article/Supplementary Material, further inquiries can be directed to the corresponding author.
